# Experimental Study and Molecular Modeling of Antibody Interactions with Different Fluoroquinolones

**DOI:** 10.3390/ijms262411862

**Published:** 2025-12-09

**Authors:** Yulia I. Meteleshko, Maria G. Khrenova, Nadezhda A. Byzova, Shen Xing, Hongtao Lei, Anatoly V. Zherdev, Boris B. Dzantiev, Olga D. Hendrickson

**Affiliations:** 1 A.N. Bach Institute of Biochemistry, Research Center of Biotechnology of the Russian Academy of Sciences, Leninsky Prospect 33, 119071 Moscow, Russiakhrenovamg@my.msu.ru (M.G.K.); nbyzova@inbi.ras.ru (N.A.B.); zherdev@inbi.ras.ru (A.V.Z.); dzantiev@inbi.ras.ru (B.B.D.); 2 Chemistry Department, M.V. Lomonosov Moscow State University, Leninskie Gory, 119991 Moscow, Russia; 3 Guangdong Provincial Key Laboratory of Food Quality and Safety, College of Food Science, South China Agricultural University, Guangzhou 510642, China; shenxing325@163.com (S.X.); hongtao@scau.edu.cn (H.L.)

**Keywords:** antigen–antibody interactions, fluoroquinolones, gatifloxacin, immunoassay, cross-reactivity, molecular modeling

## Abstract

Antibodies against low-molecular-weight compounds exhibit cross-reactivities (CRs) with their structural analogs, varying by orders of magnitude for different substances. This variability limits the informativeness of antibody applications as analytical reagents and for other aims when samples contain several members of the same family, their derivatives, or partial degradation products. Therefore, there is a demand to find some criteria for understanding the relationships between the structural characteristics of antigens of a given chemical class and their immunochemical activity. This study presents an experimental and theoretical investigation of the properties of a monoclonal antibody (MAb) against the S-stereoisomer of gatifloxacin, a member of the widely used (fluoro)quinolone (FQ) family of antibiotics, characterized by high structural diversity. The aim was to determine FQs that form complexes with MAb and suggest a methodology to predict their CRs in silico. For this, the interaction of MAb with 26 FQs was studied using the enzyme-linked immunosorbent assay and presented as CR values to the target antigen. The most pronounced CRs were observed for lomefloxacin, sarafloxacin, and ciprofloxacin. Molecular dynamics (MD) simulations were performed to identify differences in analyte interactions at the MAb antigen-binding site, which determines binding affinity. It has been shown that molecular docking fails to discriminate cross-reactive from non-cross-reactive compounds because FQs have similar cores. Therefore, advanced analysis of MD trajectories was carried out. It allowed for clarification of the dynamic features of analyte–antibody interactions responsible for binding. It was shown by the dynamical network analysis that the sum of betweenness centrality between a node corresponding to the quinolone ring and nodes representing MAb amino acids is higher for cross-reactive haptens. The found regularities can be transferred to other analyte–antibody systems as a binary classifier that discriminates cross-reactive and non-cross-reactive compounds.

## 1. Introduction

Antibodies are highly demanded bioreceptor reagents capable of binding diverse targets with high affinity and selectivity due to existing mechanisms of variability in their antigen-binding sites (paratopes). The emergence of millions of paratope variants is ensured by several factors. Among them are the diversity of genes encoding the variable regions of immunoglobulins, additional combinations of gene segments in the antibody-producing cells, mutations during the differentiation and development of these cells, and the combinatorial nature of light and heavy chain integration in the immunoglobulin molecule. Immunologists estimate the number of such variants to be in the range of 2 × 10^7^ to 10^8^. Theoretically, the possible diversity of paratope structures significantly exceeds these values. However, their actual variability is limited by the number of antibody-producing cell lines in the organism [1]. Contact with a foreign compound (antigen) entering the body activates a line of antibody-producing cells. They secrete immunoglobulins capable of binding specifically to the antigen and initiate its neutralization and elimination. These properties determine the contribution of antibodies to the immune defense of living organisms against foreign substances and pathogens. Additionally, they serve as key reagents in various analytical systems, which are increasingly applied in medical and veterinary diagnostics, biosafety, quality control of food and other consumer products, and environmental monitoring [2].

Induction of an immune response leads to the activation of several antibody-producing cell lines and, correspondingly, the generation of antibodies of variable structure. The formation of an antigen–antibody complex is ensured by the participation of dozens of amino acid residues in an immunoglobulin molecule. The achievement of high binding constants (typically in the range from 10^7^ to 10^10^ M^−1^) is the result of many non-covalent interactions—electrostatic, hydrophobic, van der Waals, and hydrogen bonding. To date, there is no possibility of rigorous a priori prediction of the paratope structure based on the antigen form, although such forecasting tools are being actively developed and improved [3,4].

The above-indicated limitations in understanding the structural basis for the formation of antigen-antibody complexes hinder the rational use of antibodies for tasks connected with their interactions with structurally similar antigens. Nevertheless, data on the presence and concentration of such antigens are essential for assessing their safety or making diagnostic conclusions. These concerns are typical and arise for the control of toxins, pharmaceuticals, hormones, and other important groups of biologically active substances [5]. Initial measurements of binding constants or cross-reactivity (CR) values for available compounds cannot cover the full variability of related antigenic molecules, including products of their biotransformation (that have undergone modification or partial destruction).

In this regard, there is a significant demand for differentiation of parameters and structural elements of antigen molecules in accordance with their impact on the formation of immune complexes. The initial idea to solve this problem was to apply the traditional concept of quantitative structural–activity relationship (QSAR) analysis [6,7,8,9]. In recent years, its prognostic capabilities have been significantly expanded via the use of computational tools for the identification of new descriptors in molecular structures (see reviews [10,11,12]).

Some recent publications demonstrate the success of such approaches. For example, Nataraj et al. used a QSAR model with more than two hundred descriptors to explore the most important sites for mutation of trastuzumab therapeutic antibody to improve its binding affinity towards its antigen, human epidermal growth factor receptor 2 [13]. In the study of Rudger et al., quantitative structure–property relationship (QSPR) modeling was used to correlate the adsorption parameters of multimodal chromatography for the purification of therapeutic antibodies [14]. More than a thousand physicochemical descriptors were used to predict the chromatographic behavior of antibodies. Simulations using the predicted adsorption parameters showed good agreement with the experimental data. In the study of Muhammad et al., machine learning-based QSAR modeling was used to predict and validate interactions of the CD33 marker of leukemia with its peptide receptor [15]. Based on the obtained results, a significant advancement in peptide-based targeted leukemia therapy was achieved, confirmed by experimental cytotoxicity studies.

Recent developments in molecular modeling tools allow for the estimation of binding energies using molecular docking approaches or various methods derived from classical molecular dynamics (MD). However, precise energy estimations, especially for a series of similar compounds, can hardly be performed due to the intricacy of biological complexes and their interactions with solvent water molecules. However, parameters other than energies can be utilized to obtain QSPR-type relations or qualitative classification [16,17]. These works introduce the idea that tight binding manifests itself not only in static interaction features but also in the dynamic behavior of the complex. If a complex is stable, the correlated motion of the inhibitor with the target protein is observed, which can be determined by dynamic network analysis. These computations are time-consuming and become possible only with the development of graphics processing unit code in MD simulations, together with recent achievements in data analysis methods.

**Figure 1:**
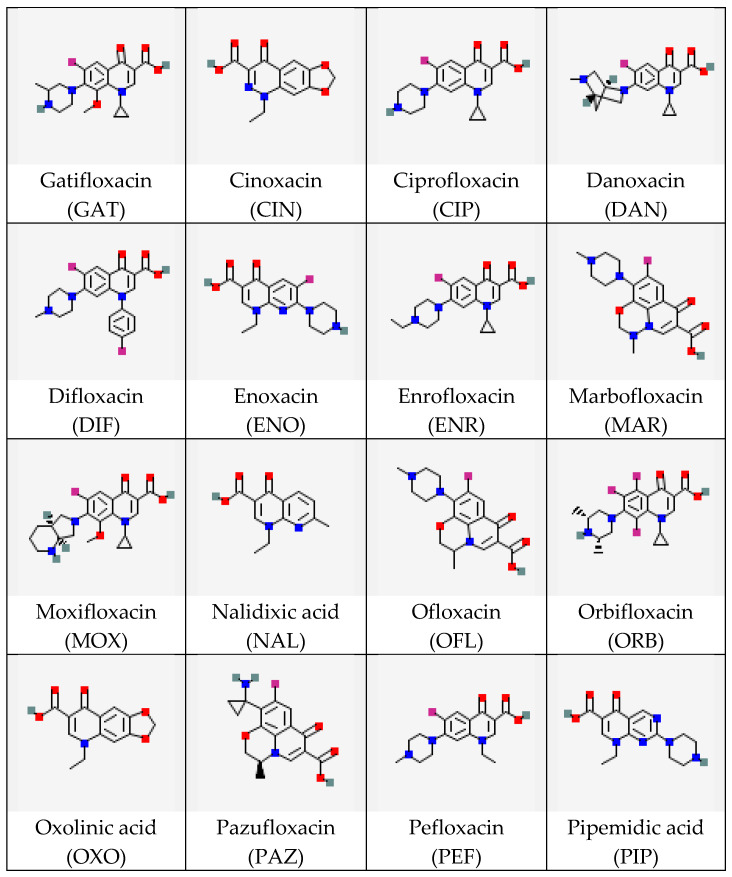

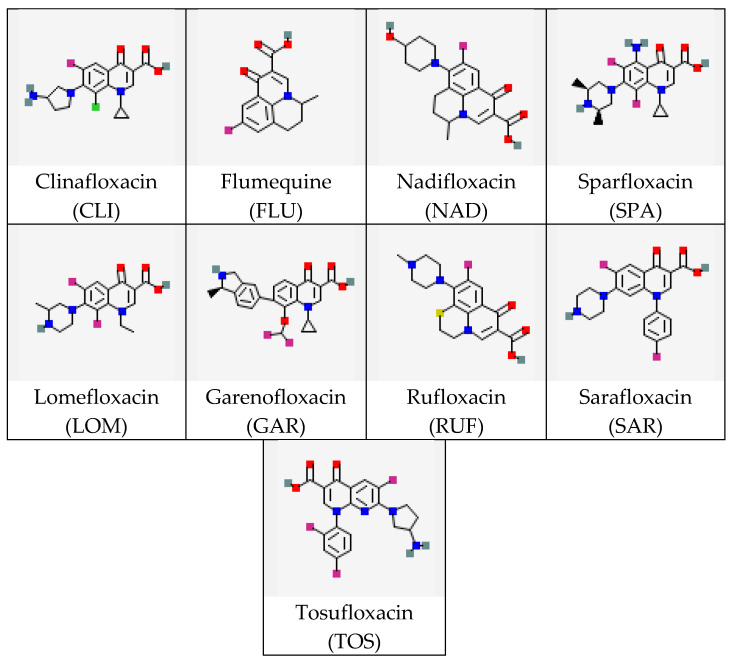
Structures of FQ molecules (from https://pubchem.ncbi.nlm.nih.gov, accessed on 3 December 2025). For optically active compounds, structures of their racemic mixtures are presented.

In this study, the interactions of a monoclonal antibody (MAb) with several (fluoro)quinolones (FQs) were considered. FQs are antibiotics active against most Gram-positive and some Gram-negative bacteria, widely used in human and veterinary medicine for the treatment of many infectious diseases [18,19,20]. The choice of FQs as objects of interest is explained by significant structural diversity and the importance of control during therapeutic measures and in assessing the quality and safety of food products of animal origin. The FQ’s molecular structure includes the quinolone ring as well as carboxyl and carbonyl groups attached at the C3 and C4 positions, respectively. The fluorine atom (if any) is located at the C6 position, while four varying radicals (at the N1, C5, C7, and C8 positions) determine the diversity of the FQ class of compounds [21]. The high structural variety of FQs necessitates the development of concepts relating typical structural parameters of their molecules to the effectiveness of interactions with specific bioreceptors, particularly antibodies. The existing pool of research on this topic (see the review [22]) does not include a comparative assessment of experimental data on antibody binding to a wide range of FQs, including stereoisomers. There is a lack of computational evaluation of the complex structures using modern high-throughput methods. Therefore, conducting such a comparison and identifying a priori unknown structural parameters key to immune binding was the objective of our study. This work includes an experimental evaluation of the selectivity of MAb specific to the levorotatory isomer of the antibiotic gatifloxacin (S-GAT) [23,24] using a wide panel of its structural analogs belonging to the same class (Figure 1).

The interactions of MAb with the target antigen and chemically similar substances were tested by using an enzyme-linked immunosorbent assay (ELISA) and presented as CR values. Based on the ELISA data, several FQs cross-reacting with anti-S-GAT MAb were identified. The dynamic network analysis of classical MD trajectories to quantify dynamic interactions between FQs and MAb was utilized. Comparison of experimental and computational data demonstrates that dynamic rather than static descriptors of interactions allow us to obtain a binary classifier of the presence/absence of CR for a set of similar compounds.

## 2. Results and Discussion

### 2.1. Study of Immune Interactions of GAT and Other FQs with Anti-S-GAT MAb

**Figure 2:**
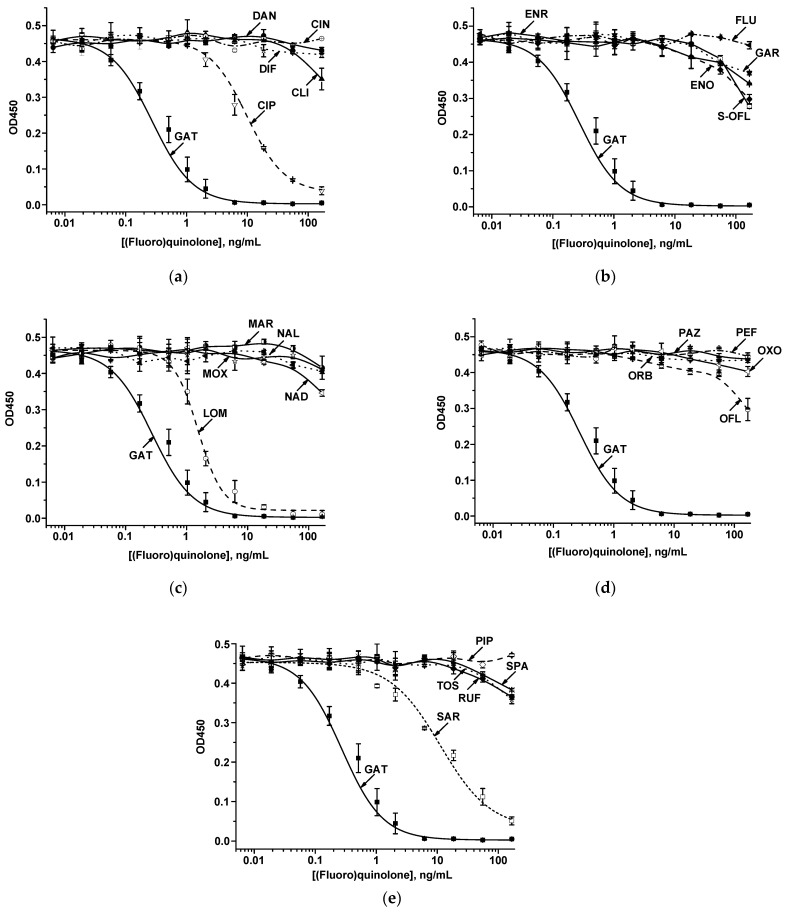
The dependencies of the competitive interactions of immobilized GAT–OVA and GAT, CIN, CIP, CLI, DAN, and DIF (**a**), GAT, ENO, ENR, FLU, GAR, and S-OFL (**b**), GAT, LOM, MAR, MOX, NAD, and NAL (**c**), GAT, OFL, ORB, OXO, PAZ, and PEF (**d**), GAT, PIP, RUF, SAR, SPA, and TOS (**e**) with anti-S-GAT specific MAb.

To study the immune interactions of FQs with S-GAT-specific MAb, the ELISA was carried out. As the conjugate adsorbed in the microplate, GAT–ovalbumin (GAT–OVA) was synthesized using the carbodiimide activation approach. Because all analytes were low-molecular-weight compounds, an indirect competitive ELISA format was applied. In this mode, a competitive interaction of free antigen in the sample and an immobilized hapten–protein conjugate with specific antibodies proceeds during the assay. The formed immune complexes are registered using anti-species antibodies (rabbit anti-goat immunoglobulins) conjugated with an enzyme label (RAGI–HRP). A panel of S-GAT structural analogs was studied for binding with MAb, namely, CIN, CIP, CLI, DAN, DIF, ENO, ENR, FLU, GAR, LOM, MAR, MOX, NAD, NAL, OFL, ORB, OXO, PAZ, PEF, PIP, RUF, SAR, S-OFL, SPA, and TOS. The CR was estimated as the ratio of the target and cross-reactive antigen concentrations at which a 50% decrease in the detected analytical signal is recorded in the competitive ELISA. As can be seen from the dependencies presented in Figure 2, anti-S-GAT MAb cross-reacted with only three compounds, LOM, CIP, and SAR (Table 1). The highest CR was established for LOM (17.5 ± 0.5%); SAR and CIP demonstrated minimal CRs (2.41 ± 0.9% and 2.74 ± 0.08%, respectively). All other FQs possessed no binding with MAb (CRs were less than 0.01%). Comparison of CRs for GAT (target hapten for antibodies) and three cross-reacting compounds (LOM, SAR, and CIP) demonstrated high reliability (*p*-values for all three pairs were <0.001 (***)).

### 2.2. Molecular Modeling

To explain the CR of some FQs to S-GAT-specific MAb, MD simulations were performed. The results of molecular docking simulations implemented for MAb and studied haptens are shown in Table 2. As can be seen, S-GAT has the lowest binding energy among all haptens (−10.0 kcal/mol). Haptens that are cross-reactive according to the ELISA (LOM, CIP, and SAR) have higher binding energies, from −8.5 to −9.7 kcal/mol. Non-cross-reactive haptens have binding energies in a wider range, lying between −5.6 kcal/mol for NAL and −9.2 kcal/mol for one of the TOS enantiomers. Evidently, because some non-cross-reactive haptens have lower binding energies than cross-reactive ones, we cannot use this descriptor to differentiate between cross-reactive and non-cross-reactive haptens.

Therefore, next, we performed classical MD simulations to discriminate haptens by their dynamic behavior. In most cases, ligands remained in the antibody binding site during trajectories. Binding site configurations for the considered systems are shown in Figure S1. As expected, S-GAT and cross-reactive haptens are stably bound to the MAb paratope. However, in only three model systems, a non-cross-reactive ligand completely left the binding site. Those are FLU *, NAL, and OXO. Interestingly, FLU * left the binding site and moved into the solution, and later returned to the protein and attached to its surface. Notably, those ligands have one of the highest binding energies in docking calculations. The other common feature is that they do not have an amino group. Most of the other haptens have an amino group oriented to the bottom of the binding site. Also, some non-cross-reactive haptens are bound not as deeply into the binding site as GAT. CIN, MAR **, and NAD * are staying closer to the solution. Overall, for most systems, ligands remain stably bound to the antibody during MD trajectories, and there are no pronounced visible differences between cross-reactive and non-cross-reactive compounds.

**Figure 3:**
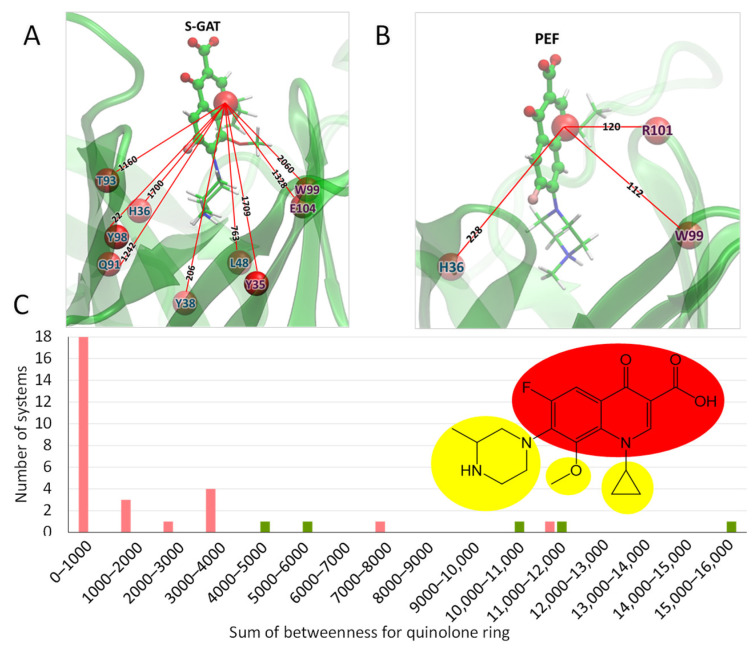
The values of betweennesses between the main node of ligand (S-GAT (**A**) or PEF (**B**)) and the amino acids of the antibody. The values have been rounded to integers. Only non-zero values are shown. (**C**) Node selection of S-GAT for dynamical network analysis and distribution of haptens by the sum of betweenness for the quinolone ring. The quinolone ring is highlighted in red. Other nodes are highlighted in yellow. Pink bars correspond to non-cross-reactive compounds, and green to cross-reactive compounds.

Hence, after that, we applied dynamical network analysis of MD trajectories to find out differences in the dynamical behavior of the investigated systems. This analysis studies the correlation of representative atoms (nodes) movements and determines if there are interactions between different parts of the system. As the descriptor, we utilized betweenness centrality, which measures the importance of a node in the whole network. We found that the sum of betweenness between a node corresponding to the quinolone ring (Figure 3A) and nodes representing amino acids of protein (Figure 3B) is higher for cross-reactive haptens and in most cases lower for non-cross-reactive ones (Table 3).

There is no strong quantitative correlation between the CR and the sum of betweenness for the FQ node. Most of the tested compounds are non-cross-reactive, and we can only define a border value for binary discrimination. This allows us to discriminate compounds that are definitely non-cross-reactive. For reactive compounds, the highest score corresponds to LOM with cross-reactivity of 17.5% and not to S-GAT, while the lower scores correspond to SAR and CIP with CRs of ~3%. The lowest score for a cross-reactive hapten is 4169. Almost all non-cross-reactive haptens fall under that score. Using 4000 as a cut-off criterion allows for the separation of non-cross-reactive haptens from cross-reactive ones, with two false positive results for DIF and ORB. The histogram in Figure 3C shows that most non-cross-reactive systems (18 from 28) are in the left part of the histogram with a betweenness score of less than 1000. We believe that the sum of betweenness scores for the quinolone ring can be used for screening purposes to discard haptens that will not be cross-reactive to S-GAT-specific MAb.

### 2.3. Consideration of Experimental (ELISA) Data

The obtained ELISA results demonstrate the high selectivity of the obtained MAb relative to other previously characterized immunoglobulin receptors against FQs. This was ensured by the proper immunogen used for MAb production based on the S-GAT hapten. Thus, Yang et al. (2024) [25] produced an anti-MAR MAb for a lateral flow immunosensor using the MAR–bovine serum albumin (BSA) immunogen. The obtained receptor was characterized by rather high CR values to OFL, ENR, NOR, and CIP (61, 32, 23, and 24%, respectively) [25]. Boonserm et al. (2021) obtained MAb to norfloxacin (NOR), which had high specificity to ENR (89%), CIP (62%), and OFL (81%), and revealed major MAb amino acid residues responsible for binding with FQs using an MD approach [26]. In the study of Acaroz et al. (2020), NOR-specific MAb produced for FQ detection in foodstuffs exhibited high CRs (from 6 to 150%) for structural analogs: 31 out of 32 tested FQs could be detected at low ppb or ppt levels [27]. Mukunzi et al. (2017) raised anti-LOM MAb for LOM detection via the ELISA and lateral flow test strip [28]. Specificity studies revealed CRs up to 112% for 6 FQs from 19 tested, including the target antigen. Nevertheless, works on highly specific MAb production can also be found in the literature [29,30].

It should be noted that in the vast majority of studies dedicated to the production of MAb to FQs, antibody specificity was stated as a fact without a theoretical analysis of the structural basis for immune interaction. In this work, we deepen the understanding of the FQ-MAb interaction mechanism. We demonstrate that the utilization of docking with simple molecular models does not lead to reliable quantities of binding affinities. This result is quite expected because the considered molecules have the same core and differ in substituents. An alternative approach based on the analysis of the dynamic behavior of FQ–MAb complexes is successfully applied to differentiate them. We propose a binary classifier of the FQs according to their ability to form complexes. As a measure of interaction, we use the betweenness centrality of the quinolone ring that defines its importance in the dynamic network formed between amino acid residues and small molecules. Thus, we demonstrate that FQs that form stable complexes effectively integrate into the dynamic network of the antibody upon binding.

## 3. Materials and Methods

### 3.1. Reagents and Materials

In the study, GAT (racemic mixture of R-GAT and S-GAT enantiomers), CIN, CIP, CLI, DAN, DIF, ENO, ENR (≥99% of purity), FLU, GAR (≥98%), LOM, MAR (≥98%), MOX (98.0–102.0%), NAD, NAL (≥98%), ofloxacin (racemic mixture of R-OFL and S-OFL enantiomers, OFL, ≥98%), ORB (≥95%), OXO, PAZ, PEF, PIP, RUF, SAR, S-ofloxacin (S-OFL, 98.0–102.0%), SPA (≥98%), TOS (≥98%), *N*-ethyl-*N’*-(3-dimethylaminopropyl)carbodiimide (EDC, ≥97%), *N*-hydroxysuccinimide (NHS, 98%), OVA (≥97%), Triton X-100 (≥99%), and dimethylformamide (DMF, ≥99.9%) from Sigma-Aldrich (St. Louis, MO, USA) were used. S-GAT (≥99%) was from DAICEL (Shanghai, China). RAGI–HRP were from Jackson Immuno Research Labs (West Grove, PA, USA). MAb against S-GAT was produced via the standard hybridoma technique using S-GAT–BSA conjugate as an immunogen [31]. A ready-to-use 3,3′,5,5′-tetramethylbenzidine (TMB)-based substrate solution was obtained from Immunotech (Moscow, Russia). All other chemicals (salts, acids, alkalis, etc.) were of analytical grade (Khimmed, Moscow, Russia). All solutions were prepared with ultrapure water with a resistivity of at least 18.2 MW (Millipore Corporation, Burlington, MA, USA).

### 3.2. Synthesis of GAT–OVA Conjugate

The GAT–OVA conjugate (the hapten:protein molar ratio was 40:1) was synthesized as a coating antigen for the ELISA as described in [32]. GAT (2.6 mg) was dissolved in DMF (0.3 mL), and then EDC (2.9 mg) and NHS (1.7 mg) were added. The mixture was stirred at room temperature for 2 h. Next, activated GAT was added dropwise to the OVA solution (7 mg) in 50 mM sodium carbonate buffer, pH 9.5 (3 mL), containing triethylamine (50 µL). The resulting reaction mixture was incubated for 2.5 h at room temperature and then for 16 h at 4 °C. The obtained conjugate was dialyzed against 50 mM phosphate-buffered saline containing 100 mM NaCl, pH 7.4 (PBS). The GAT-OVA concentration was determined spectrophotometrically at 280 nm using a Libra UV-Vis spectrophotometer (Biochrom, Cambridge, UK).

### 3.3. Enzyme-Linked Immunosorbent Assay of GAT and Other FQs

GAT-OVA (1 µg/mL, 100 µL in PBS) was immobilized in the microplate wells overnight at 4 °C. Then, the microplate was washed four times with PBS containing 0.05% Triton X-100 (PBST). After that, solutions of GAT (167–0.001 ng/mL, 50 µL in PBST) and anti-GAT MAb (0.5 µg/mL, 50 µL in PBST) were added to the wells and incubated for 1 h at 37 °C. After washing the microplate with PBST, RAGI-HRP (1:5000 dilution, 100 µL in PBST) was added to the wells and incubated for 1 h at 37 °C. After washing, the HRP activity was measured. For this, TMB-based substrate solution (100 µL) was added to the microplate wells and incubated for 10–15 min at room temperature. The reaction was stopped by adding 1 M sulfuric acid (50 µL), and the optical density (OD) at 450 nm was registered at 450 nm on a Zenyth 3100 microplate spectrophotometer (Anthos Labtec Instruments, Wals, Austria).

CR measurements were implemented using the following FQs: CIN, CIP, CLI, DAN, DIF, ENO, ENR, FLU, GAR, LOM, MAR, MOX, NAD, NAL, OFL, ORB, OXO, PAZ, PEF, PIP, RUF, SAR, S-OFL, SPA, TOS. For this, the ELISA was performed as described above using FQs as the analytes instead of GAT.

CR was calculated by the following equation:

CR = *IC*_50GAT_/*IC*_50cross-reactant_ × 100%

where *IC*_50_ is the concentration at the inflection point of the calibration curve of the detected GAT or an FQ.

### 3.4. Computational Protocol

Classical MD simulations were performed for complexes of S-GAT Fab (a part of the full-length MAb) with different haptens. For the haptens with unspecified chiral center configuration, both variants were assessed. Those haptens are LOM, NAD, CLI, TOS, and FLU. Also, pairs of stereoisomers for MAR with a chiral center at the protonated tertiary amine group and endo-exo isomers for DAN were considered. Molecular docking was used to pose ligands into the binding site. Rigid body docking was executed using the Autodock4 program [33]. Geometry optimization of hapten structures was performed in the ORCA program [34] at DFT/PBE0/6-31G** level of theory.

Coordinates of heavy atoms of S-GAT Fab were taken from PDB ID: 7F35 [31]. Hydrogen atoms were added, assuming neutral pH for charged groups. CHARMM36 force-field parameters [35] were applied for protein macromolecules, TIP3P [36] for water molecules, and CGenFF [37] for ligands. The automated procedure of charge assignment resulted in high penalties for several ligands, including CIN, DAN, MAR, MOX, and PAZ. For them, RESP charges were calculated at B3LYP/6-31G* level of theory. All minima were confirmed by the frequency analysis.

To reduce computational time, we started with benchmark simulations of two different models, one including the full S-GAT Fab and another one that was truncated. In the truncated model, all amino acids after Asp112 in the light chain and Lys118 in the heavy chain were cut off. We compared MD trajectories with full Fab and truncated Fab for S-GAT and both enantiomers, TOS and TOS*, and did not find significant differences in system behavior (Figure S2). Therefore, we proceeded with a truncated model for other ligands. All systems were solvated in rectangular water boxes so that the distances from protein surface to the box borders were not less than 15 Å. Then, they were neutralized by adding sodium or chloride ions. For all systems, we calculated 200 ns MD trajectories with a 2 fs integration step using NAMD [38]. MD simulations were performed in the NPT ensemble at T = 300 K and *p* = 1 atm.

Dynamical network analysis was performed to find out differences in the dynamical behavior of hapten–antibody complexes [39]. Every investigated system was divided into nodes—one node per amino acid and 3–4 nodes per ligand, with one of the nodes containing all non-hydrogen atoms of the quinolone ring, including halogen groups and amino group in the case of sparfloxacin. All other radicals were assigned to separate nodes. An example of the division of S-GAT into nodes is shown in Figure 3C. Betweenness matrices were calculated with the Carma program [40].

### 3.5. Statistics

All ELISA experiments were triplicated. The obtained results were expressed as means ± standard deviations. The significance of differences between the registered CRs was considered using *p*-values.

## 4. Conclusions

The extreme diversity of fluoroquinolones necessitates linking their structural features to the effectiveness of interactions with bioreceptors, particularly antibodies. Revealing the mechanistic insight into the different binding efficiencies of compounds with similar cores is a difficult task for molecular modeling. This study examined the interaction between a panel of fluoroquinolones and a specific monoclonal antibody, with a focus on the molecular recognition of structurally related compounds and their stereoisomers. The antibody was generated against the S-isomer of gatifloxacin and demonstrated a high degree of selectivity in ELISA experiments. The cross-reactivities were found only for lomefloxacin (17.5%), ciprofloxacin, and sarafloxacin (~3%), in contrast with the other 22 tested fluoroquinolones (0.01%). We demonstrate that utilization of conventional MD simulations does not discriminate between cross-reactive and non-cross-reactive compounds. On the contrary, analysis of MD trajectories allows us to suggest a binary classifier that correlates with the experimentally observed binding potency. Betweenness centrality was found to be the most efficient descriptor for estimating the binding of fluoroquinolone molecules with the antibody. For cross-reactive compounds, the betweenness centrality is higher than for non-cross-reactive compounds. This means that in the case of efficient binding, the analyte plays an important role in the dynamic network of the antibody and controls dynamic interactions between its parts. The obtained data clarify the structural background for immune recognition of fluoroquinolones with different stereoisomers. Our findings can be transferred to other non-covalent complexes with a series of related compounds to determine binding affinity.

## Figures and Tables

**Table 1:** CRs of anti-S-GAT MAb with other FQs in the ELISA.

Compound	*IC*_50_, ng/mL	CR, %
GAT	0.27 ± 0.04	100
CIN	>10,000	<0.01
CIP	9.85 ± 0.31	2.74 ± 0.08
CLI	>10,000	<0.01
DAN	>10,000	<0.01
DIF	>10,000	<0.01
ENO	>10,000	<0.01
ENR	>10,000	<0.01
FLU	>10,000	<0.01
GAR	>10,000	<0.01
LOM	1.54 ± 0.13	17.5 ± 0.5
MAR	>10,000	<0.01
MOX	>10,000	<0.01
NAD	>10,000	<0.01
OFL	>10,000	<0.01
NAL	>10,000	<0.01
ORB	>10,000	<0.01
OXO	>10,000	<0.01
PAZ	>10,000	<0.01
PEF	>10,000	<0.01
PIP	>10,000	<0.01
RUF	>10,000	<0.01
SAR	11.2 ± 4.0	2.41 ± 0.9
S-OFL	>10,000	<0.01
SPA	>10,000	<0.01
TOS	>10,000	<0.01

**Table 2:** Docking results.

Hapten	Lowest Binding Energy, kcal/mol	Mean Binding Energy, kcal/mol	Clusters, %
Cross-reactive			
S-GAT	−10.02	−9.98	100
S-LOM	−9.13	−9.06	100
R-LOM	−9.13	−9.04	100
CIP	−8.53	−8.5	100
SAR	−9.67	−9.62	100
Non-cross-reactive			
CIN	−6.1	−6.08	100
CLI	−7.84	−7.79	62
	−7.67	−7.61	38
CLI *	−8.28	−7.88	100
DAN	−8.65	−8.63	100
DAN **	−7.52	−7.47	82
	−7.47	−7.4	4
	−7.4	−7.39	14
DIF	−8.63	−8.27	85
	−8.2	−8.17	15
ENO	−8.24	−8.12	100
ENR	−8.13	−8	38
	−7.95	−7.85	17
	−7.82	−7.75	44
	−7.31	−7.31	1
FLU	−6.68	−6.68	100
FLU *	−6.49	−6.49	100
GAR	−9.05	−8.83	92
	−8.47	−8.45	7
	−8.12	−8.12	1
MAR	−8.09	−8.05	100
MAR **	−8.01	−7.88	100
MOX	−8.43	−8.31	94
	−7.58	−7.56	6
NAD	−7.79	−7.47	97
	−7.12	−7.12	3
NAD *	−7.64	−7.41	56
	−7.49	−7.42	42
	−7.11	−7.11	2
NAL	−5.59	−5.58	100
R-OFL	−7.97	−7.93	100
S-OFL	−8.04	−8.01	99
	−7.1	−7.1	1
ORB	−8.54	−8.32	100
OXO	−6.17	−6.17	100
PAZ	−8.59	−8.5	100
PEF	−7.29	−7.24	87
	−7.06	−7.06	5
	−7.03	−7.02	8
PIP	−8.23	−8.17	100
RUF	−7.45	−7.41	17
	−7.43	−7.41	64
	−7.41	−7.38	19
SPA	−7.94	−7.87	60
	−7.55	−7.49	30
	−7.37	−7.36	3
	−7.34	−7.29	7
TOS	−8.71	−8.58	100
TOS *	−9.2	−9.15	100

* for molecules with an alternative chiral center configuration at the carbon atom. ** for other molecules.

**Table 3:** Sum of betweenness for quinolone ring for all haptens, rounded to integers.

Hapten	Betweenness
S-GAT	10,189
S-LOM	15,231
R-LOM	11,178
CIP	4169
SAR	5028
CIN	450
CLI	735
CLI *	851
DAN	3252
DAN **	2167
DIF	7905
ENO	3232
ENR	462
FLU	456
FLU *	0
GAR	710
MAR	462
MAR **	462
MOX	1362
NAD	1062
NAD *	882
NAL	0
R-OFL	3227
S-OFL	211
ORB	11,033
OXO	0
PAZ	1840
PEF	460
PIP	3339
RUF	644
SPA	858
TOS	628
TOS *	462

* for molecules with an alternative chiral center configuration at the carbon atom. ** for other molecules.

## Data Availability

The original contributions presented in this study are included in the article/Supplementary Material. Further inquiries can be directed to the corresponding author.

## Supplementary Materials

The following supporting information can be downloaded at https://www.mdpi.com/article/10.3390/ijms262411862/s1.
