# Supplementary material for: Experimental Study and Molecular Modeling of Antibody Interactions with Different Fluoroquinolones
Source: Int J Mol Sci. 2025 Dec 9;26(24):11862. doi: 10.3390/ijms262411862 (PMC12733293; doi:10.3390/ijms262411862)
Supplement: Supplementary file 1 [file ijms-26-11862-s001.zip › ijms-4004950-SI.pdf]

SUPPLEMENTARY INFORMATION

Experimental Study and Molecular Modeling of Antibody Interactions with Different Fluoroquinolones

Yulia I. Meteleshko [1,2], Maria G. Khrenova [1,2], Nadezhda A. Byzova [1], Shen Xing [3], Hongtao Lei [3], Anatoly V. Zherdev [1], Boris B. Dzantiev [1], and Olga D. Hendrickson [1,*]

1 A.N. Bach Institute of Biochemistry, Research Center of Biotechnology of the Russian Academy of Sciences, Leninsky Prospect 33, 119071 Moscow, Russia; nbyzova@inbi.ras.ru (N.A.B.); zherdev@inbi.ras.ru (A.V.Z.), dzantiev@inbi.ras.ru (B.B.D.), odhendrick@gmail.com (O.D.H.)
2 Chemistry Department, M.V. Lomonosov Moscow State University, Leninskie Gory, 119991 Moscow, Russia; meteleshko.yulia@gmail.com (Y.I.M.), khrenovamg@my.msu.ru (M.G.K.);
3 Guangdong Provincial Key Laboratory of Food Quality and Safety, College of Food Science, South China Agricultural University, Guangzhou 510642, China; shenxing325@163.com (S.X.); hongtao@scau.edu.cn (H.L.)
* Correspondence: odhendrick@gmail.com; Tel.: +7-495-954-28-044

Figure S1 presents alignments of binding site configurations from the end of MD trajectories between S-GAT and other considered molecules. Cross-reactive haptens have slightly different positions in the binding pocket from S-GAT. All non-cross-reactive systems are divided into two groups. The first group consists of systems in which ligands have similar positions in the binding pocket to S-GAT and cross-reactive haptens. The second group consists of all other systems, including those with ligands bound less deeply into the binding pocket or ligands that have a strongly tilted quinolone ring compared to S-GAT and cross-reactive haptens.

S-GAT and cross-reactive haptens

Academic Editor: Firstname Lastname

Received: date
Revised: date
Accepted: date
Published: date

Citation: To be added by editorial staff during production.

Copyright: © 2025 by the authors. Submitted for possible open access publication under the terms and conditions of the Creative Commons Attribution (CC BY) license (https://creativecommons.org/licenses/by/4.0/).

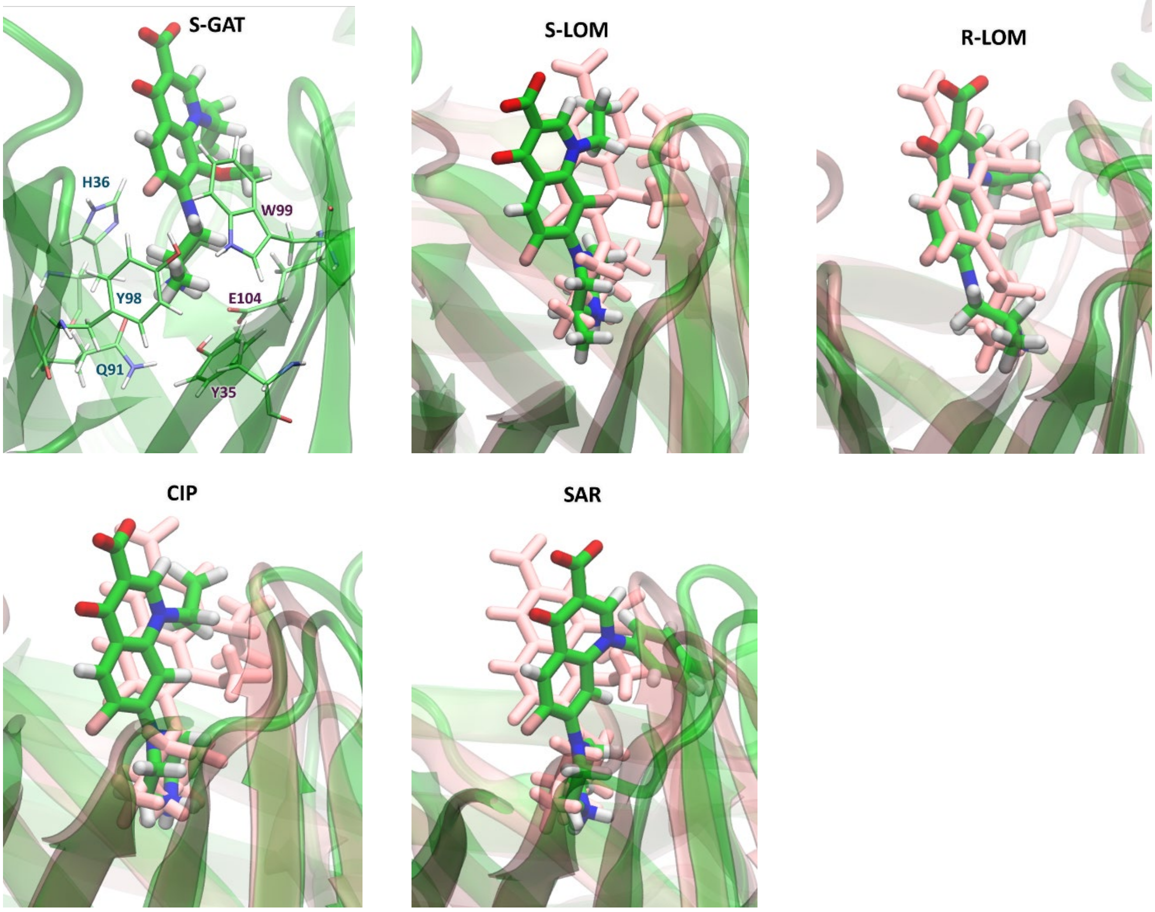

Non-cross-reactive haptens which binds similarly to S-GAT according to MD simulations

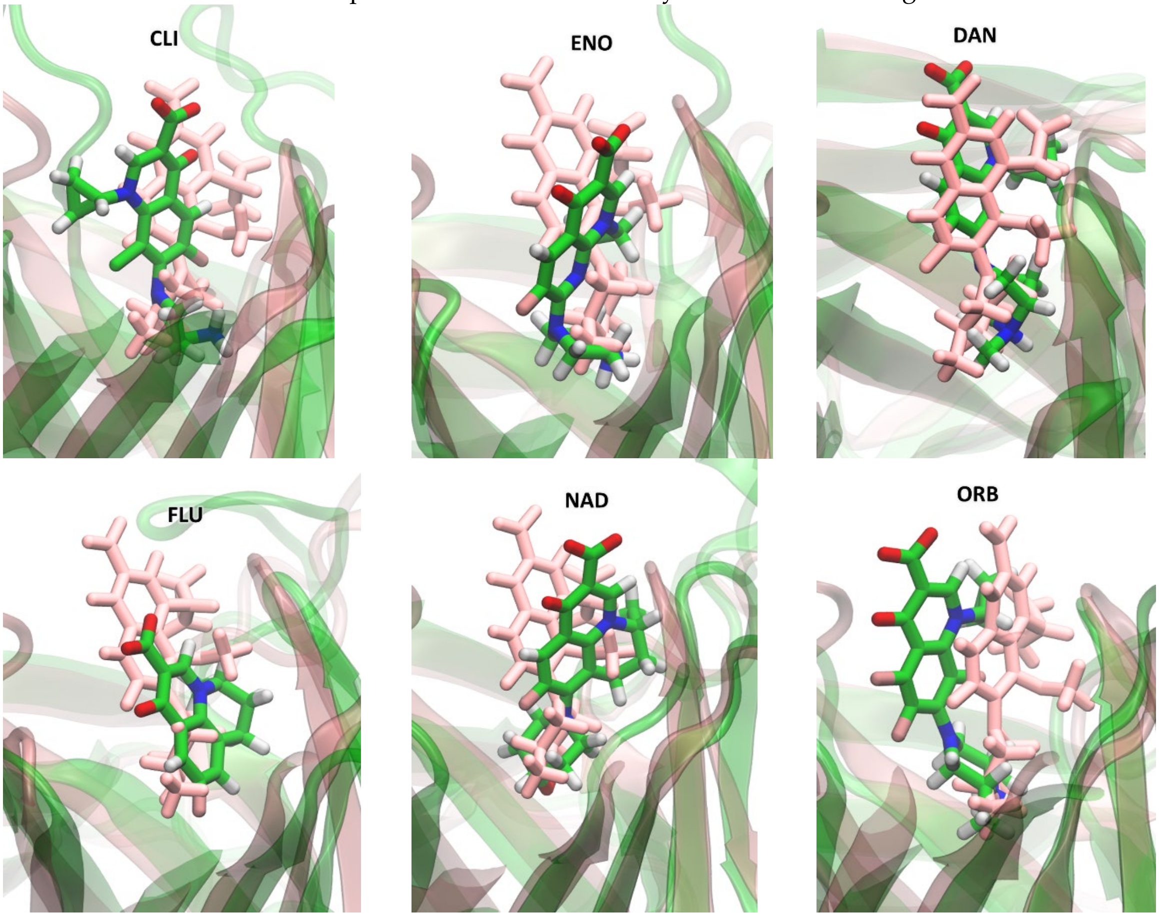

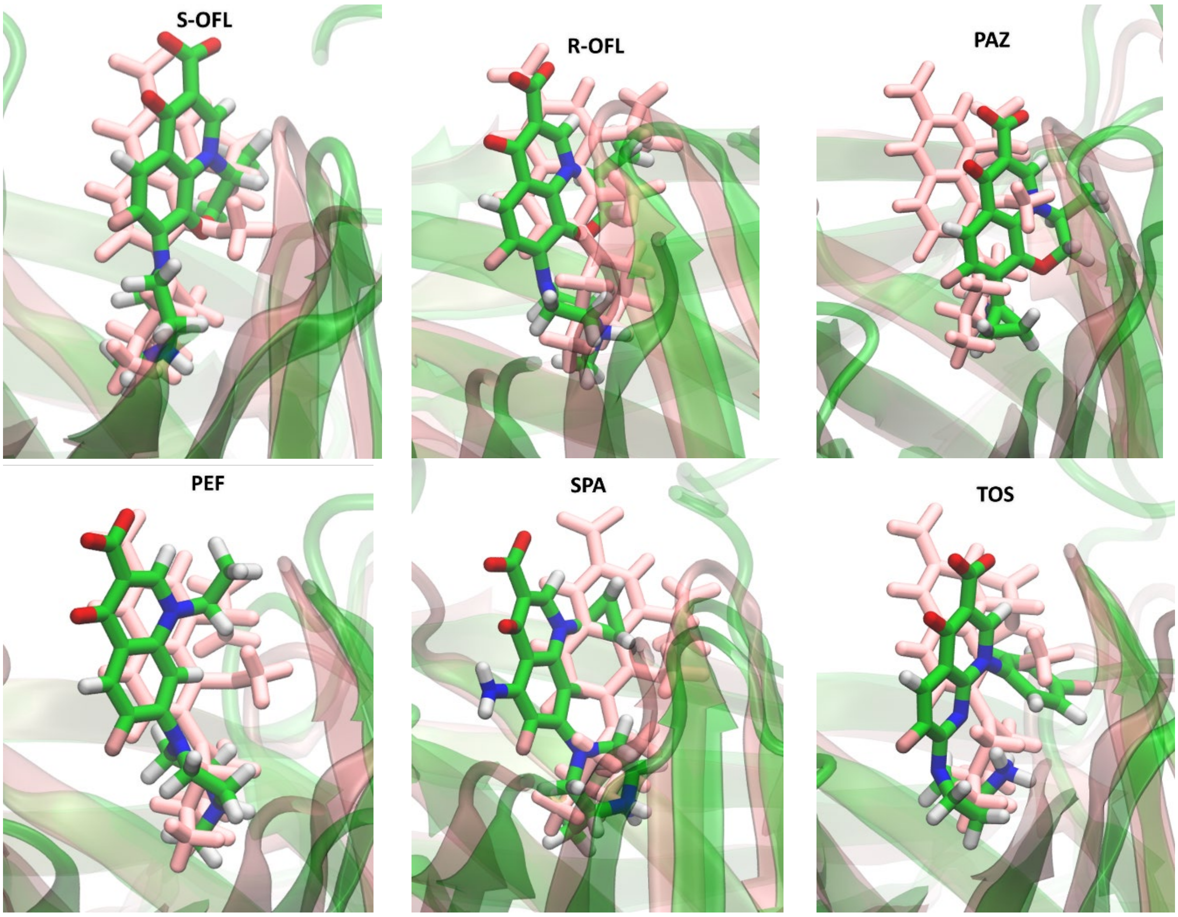

Other non-cross-reactive haptens

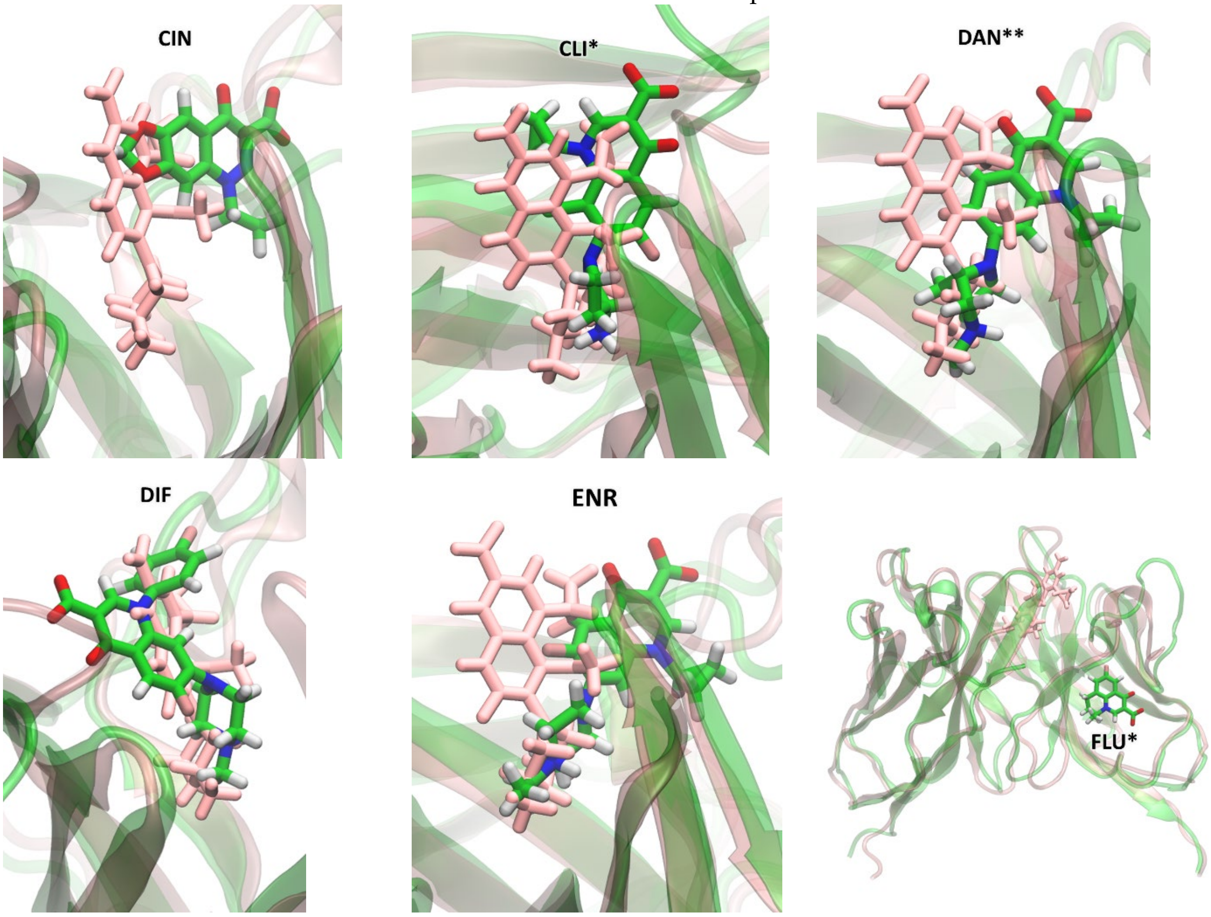

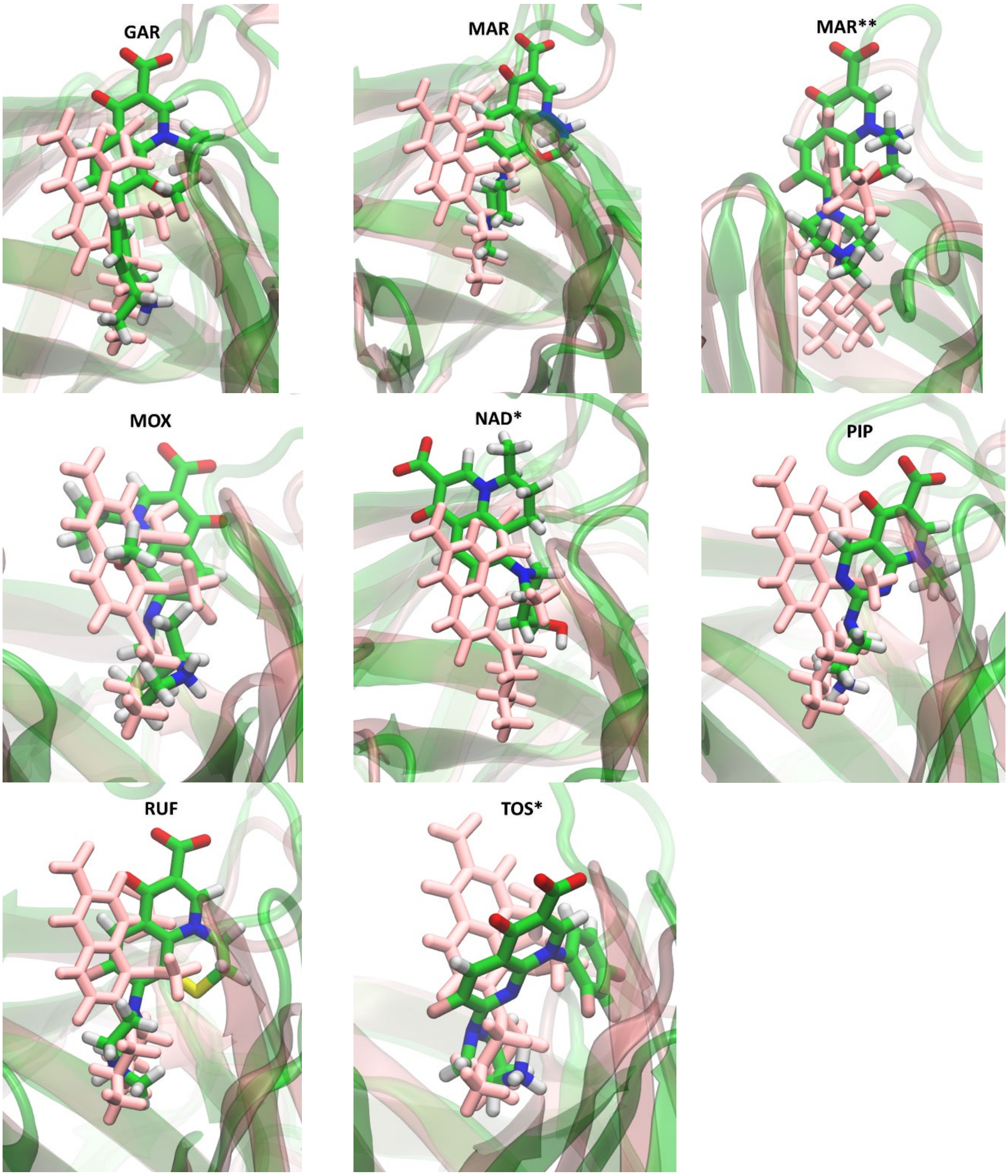

Figure S1. Binding site configurations at 200 ns for the considered model systems. For NAL and OXO, there are no images because those ligands remain in the binding site. After leaving the binding site, FLU* stayed at the surface of the protein. Haptens are drawn in bold sticks. In the picture for S-GAT, important amino acids from the binding site are drawn in thinner sticks. Blue and violet fonts are used for amino acids in light and heavy chains of the antibody, respectively. For all other haptens, the S-GAT position is shown in light pink sticks.

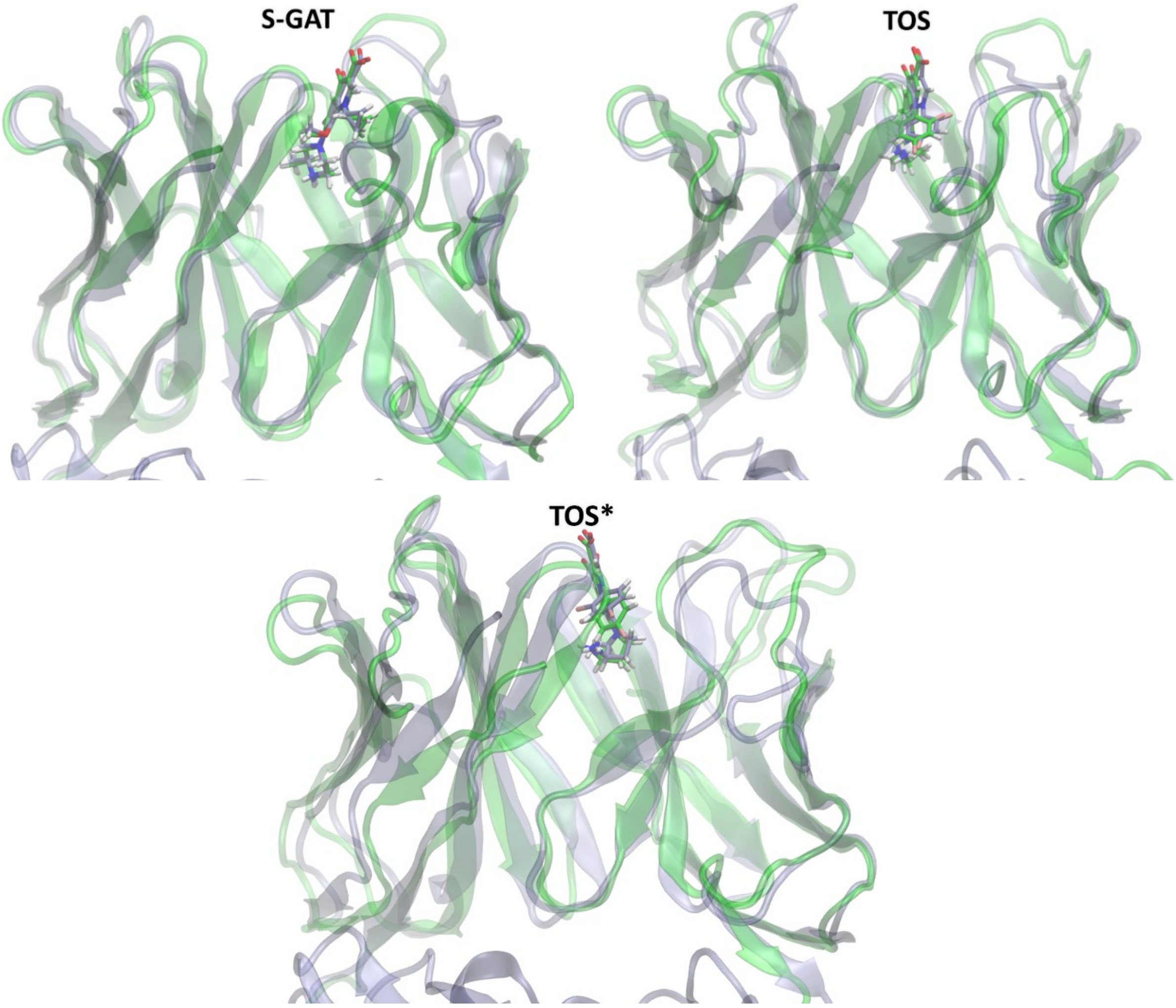

Figure S2. Full and short Fab models at 200 ns.
